# Application of geographically-weighted regression analysis to assess risk factors for malaria hotspots in Keur Soce health and demographic surveillance site

**DOI:** 10.1186/s12936-015-0976-9

**Published:** 2015-11-18

**Authors:** Mansour M. Ndiath, Badara Cisse, Jean Louis Ndiaye, Jules F. Gomis, Ousmane Bathiery, Anta Tal Dia, Oumar Gaye, Babacar Faye

**Affiliations:** Service Parasitologie, Université Cheikh Anta Diop, Dakar, Senegal; London School of Hygiene and Tropical Medicine, London, UK; Institut de santé et de développement, UCAD, Dakar, Senegal; Unité Mixte Internationale « Environnement, Santé, Sociétés » (UMI3189 ESS), CNRS-UCAD-CNRST-USTTB-UGB, Dakar, Senegal; Centre de Suivi Ecologique, CSE, Dakar, Senegal

**Keywords:** Malaria, Hotpots, Geographically-weighted regression, Disease mapping, Spatial analysis, OLS

## Abstract

**Background:**

In Senegal, considerable efforts have been made to reduce malaria morbidity and mortality during the last decade. This resulted in a marked decrease of malaria cases. With the decline of malaria cases, transmission has become sparse in most Senegalese health districts. This study investigated malaria hotspots in Keur Soce sites by using geographically-weighted regression. Because of the occurrence of hotspots, spatial modelling of malaria cases could have a considerable effect in disease surveillance.

**Methods:**

This study explored and analysed the spatial relationships between malaria occurrence and socio-economic and environmental factors in small communities in Keur Soce, Senegal, using 6 months passive surveillance. Geographically-weighted regression was used to explore the spatial variability of relationships between malaria incidence or persistence and the selected socio-economic, and human predictors. A model comparison of between ordinary least square and geographically-weighted regression was also explored. Vector dataset (spatial) of the study area by village levels and statistical data (non-spatial) on malaria confirmed cases, socio-economic status (bed net use), population data (size of the household) and environmental factors (temperature, rain fall) were used in this exploratory analysis. ArcMap 10.2 and Stata 11 were used to perform malaria hotspots analysis.

**Results:**

From Jun to December, a total of 408 confirmed malaria cases were notified. The explanatory variables-household size, housing materials, sleeping rooms, sheep and distance to breeding site returned significant t values of −0.25, 2.3, 4.39, 1.25 and 2.36, respectively. The OLS global model revealed that it explained about 70 % (adjusted R^2^ = 0.70) of the variation in malaria occurrence with AIC = 756.23. The geographically-weighted regression of malaria hotspots resulted in coefficient intercept ranging from 1.89 to 6.22 with a median of 3.5. Large positive values are distributed mainly in the southeast of the district where hotspots are more accurate while low values are mainly found in the centre and in the north.

**Conclusion:**

Geographically-weighted regression and OLS showed important risks factors of malaria hotspots in Keur Soce. The outputs of such models can be a useful tool to understand occurrence of malaria hotspots in Senegal. An understanding of geographical variation and determination of the core areas of the disease may provide an explanation regarding possible proximal and distal contributors to malaria elimination in Senegal.

## Background

Malaria is one of the major diseases that contributed to health problems worldwide. There were an estimated of 207 million cases of malaria worldwide in 2012 and most of the estimated cases (80 %) occurring sub-Saharan Africa [1]. Two main factors are largely responsible for the deaths attributed to malaria in sub-Saharan Africa. First, the majority of infections in this region are caused by *Plasmodium falciparum*, the most dangerous of the four human malaria parasites and secondly the most efficient mosquito vector, *Anopheles gambiae,* is widespread in the region and is very difficult to control [2]. The species has a long life expectancy, strong anthropophagy, and high abundance, which can lead to several hundred secondary malaria cases from a single infected individual [3, 4].

The disease remains an important public health problem in Senegal, where it is mostly seasonal with its major incidence during the rainy season. Recent data from the National Malaria Control Programme (NMCP) indicate that malaria is endemic in more than 26 health districts, with an incidence rate greater than 25 per 1000 inhabitants in some parts of the country.

In Senegal, considerable efforts had been made to reduce malaria morbidity and mortality during the last decade. This had resulted to a marked decrease of malaria cases. With the decline of malaria cases, transmission has become sparse in most of the Senegalese health districts.

In recent years, there has been a growing interest among the Ministries of Health through the NMCP in the use of geographical information systems (GIS) as a tool to strengthen the analytical, management, monitoring and decision-making capacity in public health, as well as a tool for advocacy and communication between technical personnel, policy makers and the general public. This is a result of the recognition of the capacity of GIS in managing geographical dimensions, integrating health-related data from various sources, helping to discover and visualize new patterns and geographical relations in data that would otherwise be difficult to identify, and displaying these on maps that constitute a more expressive and visual representation. Recognizing the power of this tool (GIS) has led to a growing number of health studies and projects being developed by academic teams and health service professionals that include its use as a tool for identifying and characterizing malaria hotspots. The dependence of malaria transmission on its spatial and ecological context has long been recognized; hence, the need to study malaria disease within its explicit spatial context [5, 6]. GIS has been widely applied to the understanding and management of malaria in Africa. For example GIS has been used to generate models of malaria occurrence [7, 8], seasonality [9, 10] and transmission intensity [11–16] using climatic and remotely-sensed data.

This study was undertaken in Keur Soce health and demographic surveillance site (KSHDSS). It is located in Ndoffane health district. This study investigates malaria hotspots in Keur Soce sites by using geographically-weighted regression. Because of the occurrence of hotspots, spatial modelling of malaria cases could have a considerable effect in disease surveillance (especially in malaria control) in Senegal towards the certain reduction (if not complete elimination) of malaria infection and deaths.

## Methods

### Study area and population

The primary tool of health and demographic surveillance is a rigorous annual update of the demographic status of every member of geographically defined population, namely the Keur Soce Sub District of the Ndoffane District. This comprises 74 villages. A baseline census was conducted in 2010. Since then, updates have been conducted, collecting information on all births, deaths and in-and-out-migration in the surveillance population. A field operation was performed each year to visit each of the almost 3000 households in the sub-district interviewing the best respondent available, who must be adequately knowledgeable of the status of household events. During this interview, the fieldworker verified existing records, recorded new data pertaining to individuals or the household and recorded the demographic events that have occurred since the preceding year’s census update. Enquiry into the demographic event experienced by each household members were supplemented by a full maternity history of all in-migrant women aged 15–55 years, as well as residence histories, and other modules built into the census. The census update was conducted by two census teams of five fieldworkers each with supervisor who scrutinized GIS-based maps listing every dwelling in the area. The maps were kept up to date by taking GPS (Global Positioning System) readings of new dwellings each year.

A verbal autopsy was conducted on each death to establish the cause. The verbal autopsy interview was conducted by a trained lay fieldworker in the vernacular i.e. Wolof, and assessed by medical practitioners to establish the main cause of death, as well as immediate and contributing causes. In this way, a longitudinal database of demographic events has been established and updated during each round.

**Fig. 1:**
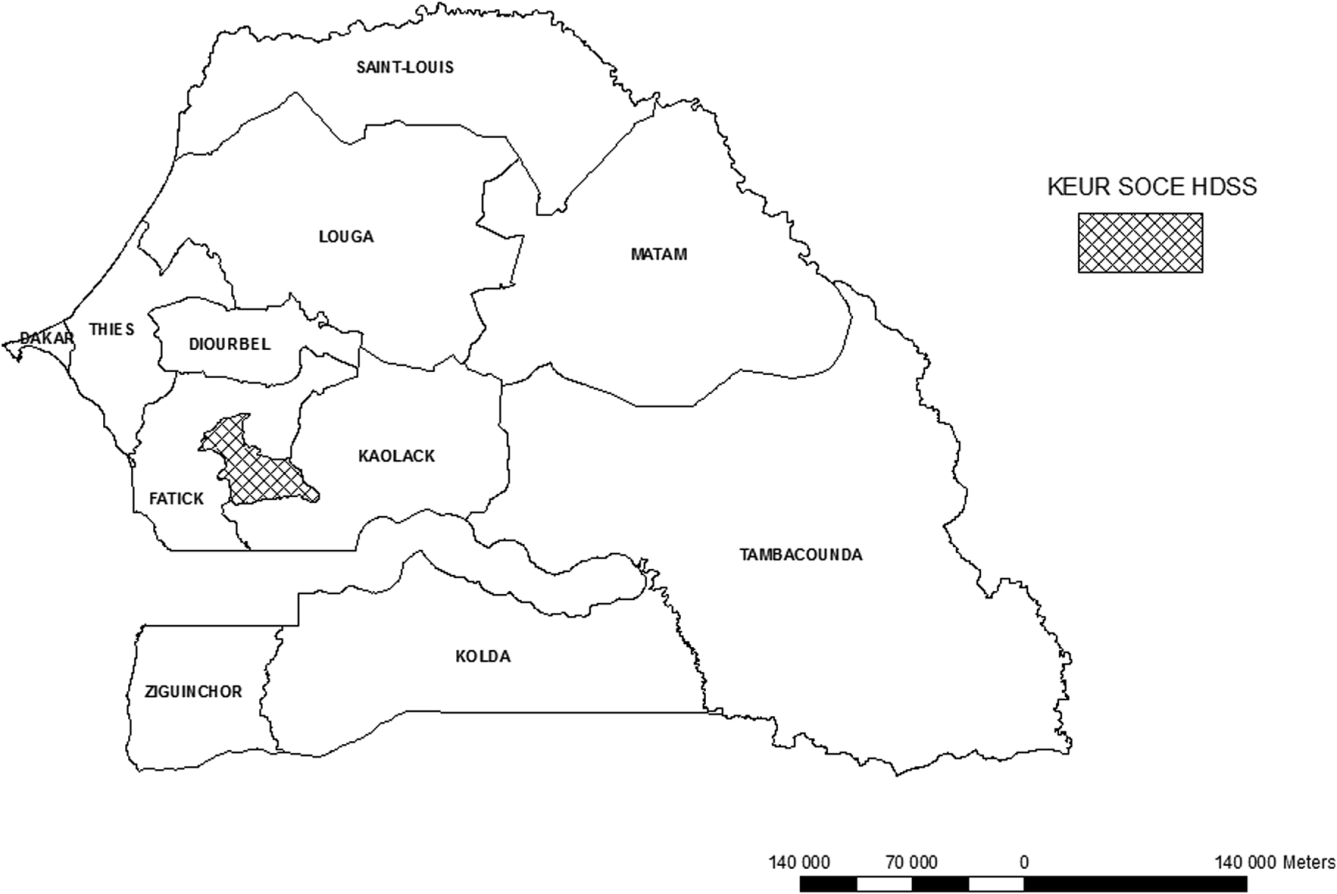
Localization of Keur Soce HDSS in Senegal

The Keur Soce Health and Demographic Surveillance Site (KSHDSS) is located in rural area in the region of Kaolack, in the district of Ndoffane. The area lies between longitudes 16°00′14.8″ and 16°07′13″W and latitudes 13°51′53″ and 14°00′00″N. It is located at 230 km from Dakar in the Sudano-Sahelian region of Senegal (Fig. 1).

Regarding its localization, the site’s ecology is characterized by the alternation of a long and dry season from November to June and a short rainy season from July to October. The population of the Keur Soce HDSS in August 2013 was 32,601, which is less than 1 % of the total population of the country and about 2 % of the total population of Kaolack’s region. Demographic monitoring covers 74 villages of varying size. The main religious faith is Islam, with more than 97 % of muslims. The main language spoken is Wolof.

In addition, lack of communication systems, road network and electricity within the health district also affects the health of the population. There are two health posts and 15 health huts operating within the study area that are providing basic services to the study population. These include curative care, immunization, prenatal care, delivery, and oral rehydration therapy and malnutrition management. The residential unit is the compound, which consists of one or more households together with some members of the extended patrilineal family. Traditional houses are huts (one for each couple that is married and an additional huts for young unmarried people). In most of the villages, there are some modern constructions made with cement walls and iron roofs. Water from taps and fountains are the main source of drinking water for the population. Most of the villages have electricity.

Subsistence agriculture is the mainstay of the district’s economy, complemented to some extent by retail trading. About 95 % of the people are farmers. The major agricultural products are groundnuts, millet, maize, beans. Rearing of cattle, goats, sheep and fowls also form part of the agricultural activities. Unfortunately, the rainfall pattern limits food cultivation to a single growing season.

The district has 10 primary schools, and one secondary school in Keur Soce village. In each of the village, there is a “DAARA”, i.e. an Islamic school, where children learn the Koran and arabic.

### Passive surveillance of malaria

From June to December 2013, patients self-presenting at the health structures with fever or a history of fever in last 24 or 48 h are normally diagnosed at the health post and the health huts in Keur Soce health and demographic surveillance site system. For each patient the government health workers routinely perform a rapid diagnostic test (RDT) on a finger prick blood sample to diagnose malaria, and record basic details in a register.

### Outcome definition

To analyse the spatial relationships between malaria occurrence and socio-economic and environmental factors in small communities in Keur Soce, Senegal, the confirmed malaria cases during the 6 months of passive surveillance were used. This was defined as any resident (any person who has been staying at least 3 months in the study area) self-presented at the health structures with fever or history of fever in last 24 or 48 h and had been diagnosed malaria positive by RDT and confirmed by microscopy.

### Data collection

Blood film for “gold standard” microscopy confirmation of the RDT test (which was done in parallel with the finger-prick sample thus avoiding the need for repeated sampling) was performed and an individual questionnaire was used to investigate determinant factors at the household level.

GIS coordinates were measured at the household level. The exposures of interest are the individual use of protective measures, and the coverage of control measures at the community level and household level. This will help to understand the direct and indirect effects of these measures. The analysis included also demographic and socioeconomic variables, to understand the population groups who are now at risk. Each patient was visited at home to record current ITN use (based on inspection of the place where the patient sleeps, net type and condition, and net use); of other interventions [including other antivectorial measures, and the timing of any intermittent preventive treatment (IPT) doses]. Coverage of ITNs and other protective measures in the vicinity of the person’s home; proximity to mosquito breeding sites were investigated. For children less than 10 years vaccination status were assessed with the vaccination card.

### Data management and cleaning

Data were double-entered in an Access 2007 database, checked for errors or inconsistencies and analysed. The localization (longitude and latitude) of all households were recorded using eTrex Venture single handheld GPS receivers. Administrative boundary data were obtained from the “*Centre de Suivi Ecologique*” (CSE) of the Government of Senegal. GPS records were imported in ArcMap 10.2 software and checked on the polygon boundary map. All errors were checked at field level. Distances between points of interest (distance from households to every health facility) were calculated using ‘costed distance surface’.

### Statistical analysis

**Fig. 2:**
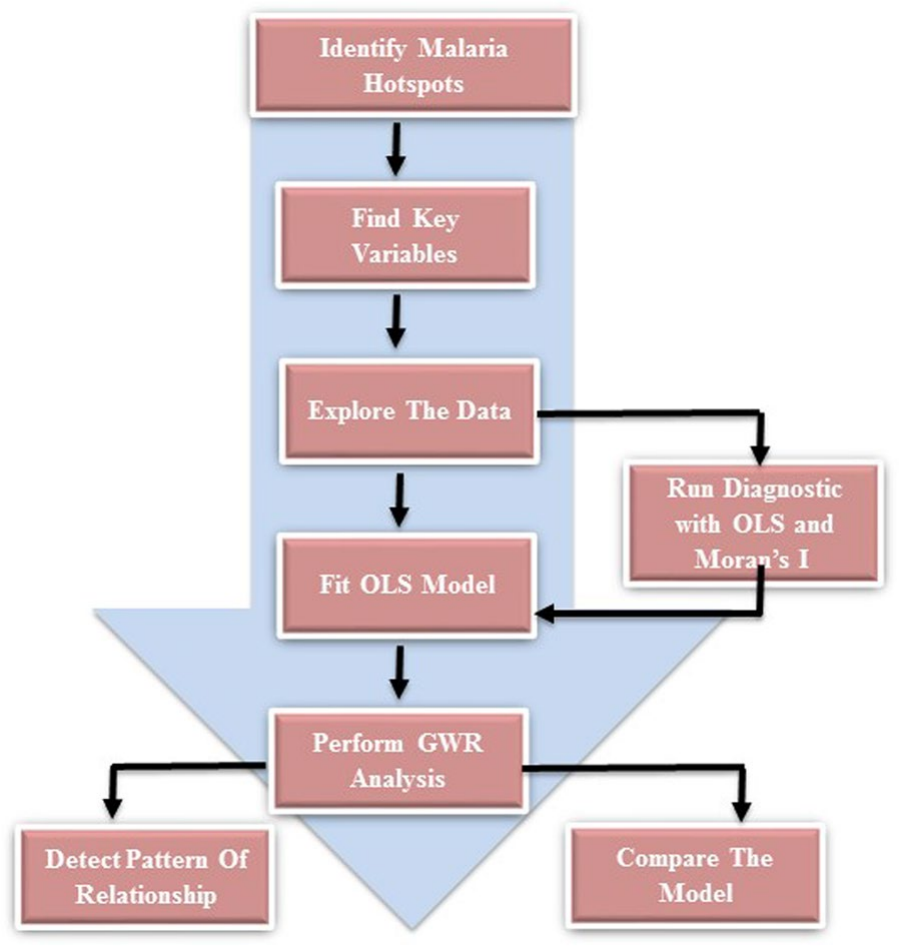
Methodological framework

**Fig. 3:**
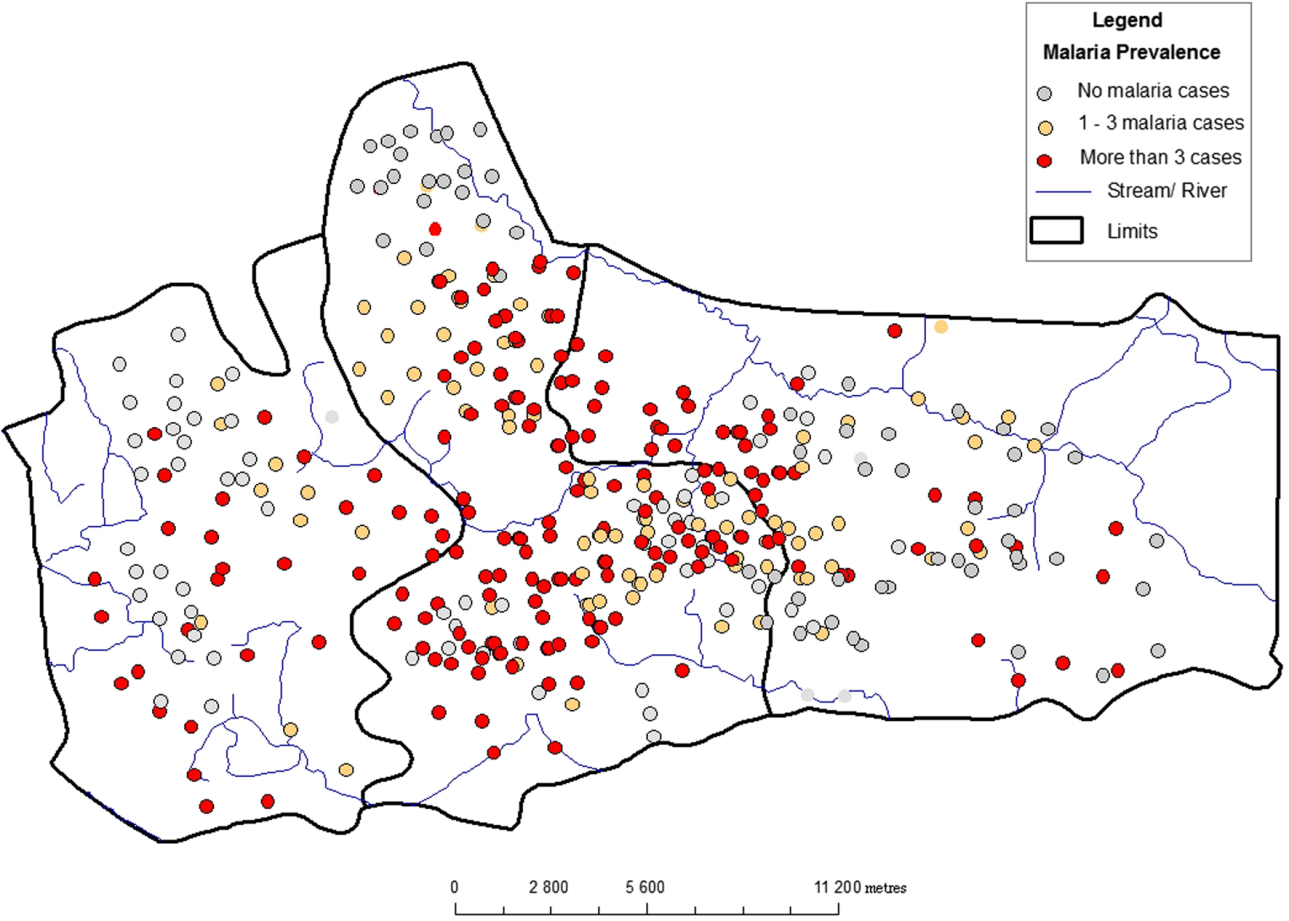
Spatial distribution of household with and without RDT positive

**Fig. 4:**
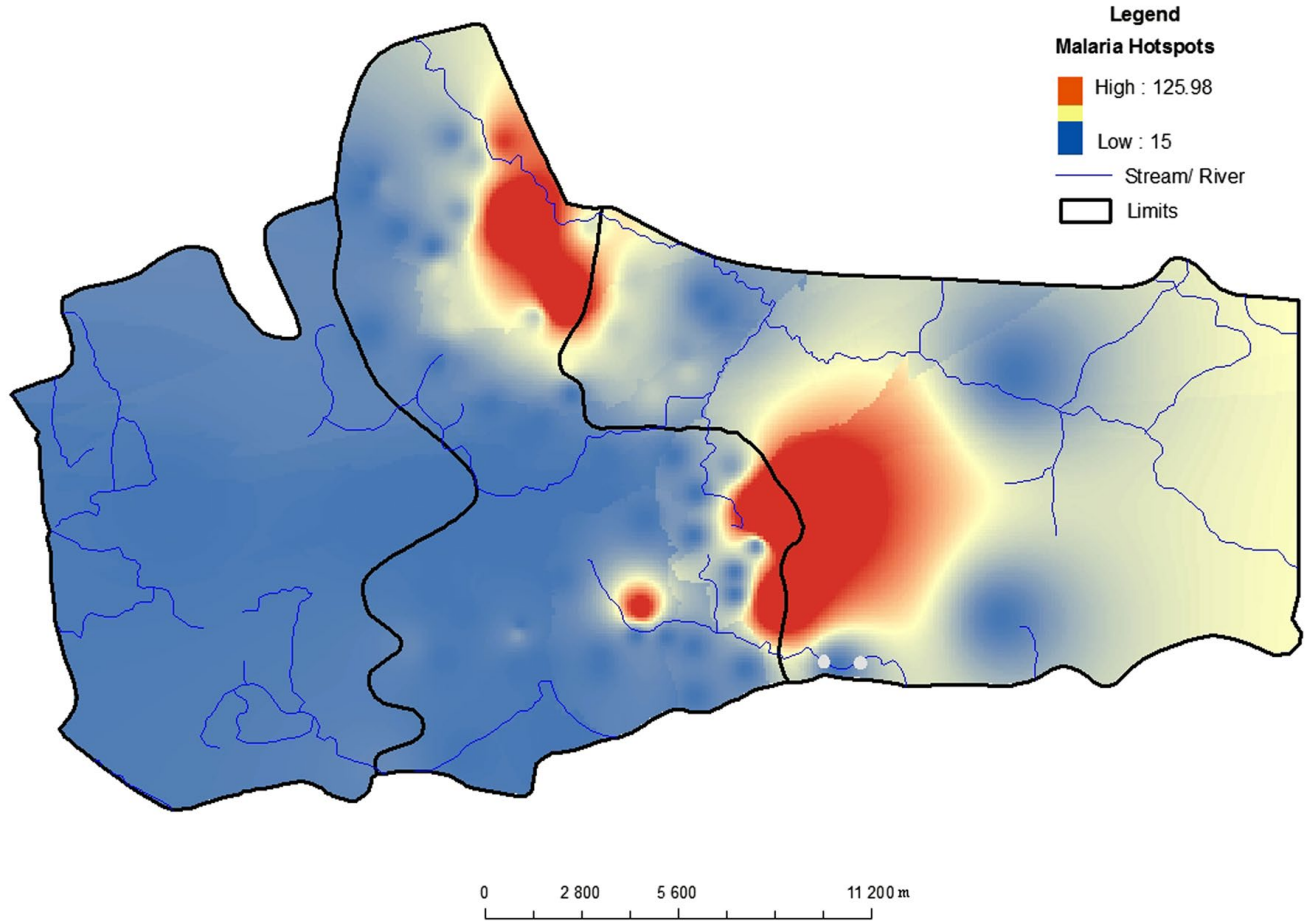
Malaria hotspots in Keur Soce HDSS

The basic software used for computation, exploratory analysis, mapping and visualization is ArcMap version 9.2. This GIS software was chosen because it presents numerous extensions for spatial statistical and geostatistical modelling (such as GWR, spatial autocorrelation and other geostatistical analyst tools). Generally, these techniques were used to map spatial pattern, test relationships, check for redundancy among the explanatory variables and geo-visualization. The model’s framework is shown in Fig. 2. The dependent variable for this model is the confirmed malaria cases from June to December 2013 by village level. These statistical values were entered into the prepared GIS vector polygon map as non-spatial data. To visualize the spatial distribution of such data, a choropleth map was generated to show the prevalence of malaria within household in the study area. For each household a number of confirmed malaria cases during the passive surveillance was geolocalized and mapped. Figure 3 shows the malaria prevalence within household. For the classification, three classes were produced: households with no confirmed cases, households with one to three confirmed cases and households with more than three cases. In order to detect malaria hotspots and show continuous distribution, empirical Bayesian kriging model with log empirical data transformation method was applied on the map Fig. 4.

Basically, the first fundamental geographic question (the where question) regarding malaria prevalence in the study area has been answered by Fig. 4 (i.e. by displaying the location of malaria hotspots and the spatial pattern of distribution). The next logical geographic questions that follow are “why” such clustering pattern. And “what” are the likely factors that are associated with this observed pattern? The GWR is designed to answer such scientific questions and others like, does the relationship between the dependent variable and the predictors varies across space. Which explanatory variable shows stronger influence in a certain area?

Several socio-economic, demographic and environmental factors were identified and selected for the analysis as explanatory variables. Population density was included as a predictor variable because it may exert strong influence over malaria occurrence and spread (i.e. it is expected that malaria cases would be high in high population density areas).

### Modeling spatial relationship

OLS and GWR spatial statistic were employed for exploring the spatial relation between malaria occurrence and the selected explanatory variables (Table 1). The linear regression was used as a diagnostic tool and for selecting the appropriate predictors (with respect to their strength of correlation with the criterion variable) for the GWR model.

**Table 1:** Candidate explanatory variables

Variables	Values
Age	0–69
Gender	0–100 % for both males and females
Household size	1–35
Village size	10–3698
Sleeping rooms	1–9
Bed net use	1–25
Distance to health Facilities	1–15
Temperature	18.1–41.7
Raining days	15–39
Housing materials	Material used for walls, roof, and floor
Cow	2–125
Goat	1–66
Sheep	0–99
Donkey	0–58
Horses	0–15
Distance to breeding site	10–300

The multicollinearity was assessed with the variance inflation factor (VIF). This is defined by the equation:

$$VIF_{j} = \frac{1}{{1 - r_{j}^{2} }}$$

where $r_{j}^{2}$ is the coefficient of determination when variable Xj is regressed on the j − 1 remaining independent variables. If the VIF value(s) is greater than 10, it therefore indicates the existence of multicollinearity among the predictors. In addition, autocorrelation statistic was applied to detect whether there is spatial autocorrelation or clustering of the residuals, which violate the assumption of Poisson regression. Progressively, the spatial independency of the residuals was assessed with the global spatial autocorrelation coefficient Moran’s I. This is defined by the equation:

$$I = \frac{{N\sum\limits_{{{\text{i}} = 1}}^{\text{n}} {\sum\limits_{{{\text{j}} = 1}}^{\text{n}} {{\text{w}}_{\text{ij}} \left( {{\text{x}}_{\text{i}} - {\bar{\text{x}}}} \right)\left( {{\text{x}}_{\text{j}} - {\bar{\text{x}}}} \right)} } }}{{\left( {\sum\limits_{{{\text{i}} = 1}}^{\text{n}} {\sum\limits_{{{\text{j}} = 1}}^{\text{n}} {{\text{w}}_{\text{ij}} } } } \right)\sum\limits_{{{\text{i}} = 1}}^{\text{n}} {\left( {{\text{x}}_{\text{i}} - {\bar{\text{x}}}} \right)^{2} } }}$$

where: N is the number of observations (points or polygons), ${\bar{\text{x}}}$ is the mean of the variable, X_i_ is the variable value at a particular location, X_j_ is the variable value at another location, W_ij_ is a weight indexing location of i relative to j.

Moran’s I values ranges from +1 (positive autocorrelation) and −1 (negative autocorrelation). The expected outcome in this case is a complete random pattern i.e. no spatial autocorrelation.

Geographically weighted regression (GWR) has been developed as an extension of traditional regression to incorporate, detect, and account for spatial non-stationarity in variable relationships in the model [17]. This spatially localized model assumes that relationships between regression variables may vary over space; consequently, it generates a set of local line regression models rather than a global model, with estimates for every sample in space. A moving window approach allows the weights of each spatial unit to vary as a function of the spatial relationship between them. Namely, a local estimation of model parameters is derived by using a subsample of data from nearby observations, which are weighted by using a decreasing function of distance. In this way, the impacts of neighboring samples are stronger than those farther away. A threshold, called the kernel bandwidth, is specified to indicate the distance beyond which neighbours no longer have influence on local estimates. A geographic surface of models is derived with associated goodness-of-fit statistics and localized parameter estimates such as R -square, standard error, and t values. These maps highlight possible data relationships, aid finding exceptions or local hotspots, and provide information on the nature of the processes under study. GWR coefficient values was used to explore the spatial variability of relationships between malaria incidence or persistence and the selected, socio-economic and human predictors. In order to determine the optimal bandwidth of the kernel function, the Akaike Information Criterion (AIC) was applied.

### Ethical considerations

This study was reviewed and approved by the Senegalese Ministry of Health through the National Ethics Committee. All participants signed an informed consent before being enrolled in the study and handled a signed or marked with a fingerprint informed consent. All household heads signed the consent form.

## Results

### Clinical and demographic profile of malaria patient

Table 2 presents the socio-demographic and clinical characteristics of the study population. Out of a total of 764 patients diagnosed for malaria, 408 were treated as malaria confirmed cases and 356 cases were malaria free patient.

**Table 2:** General characteristics of malaria cases

Factors	Malaria		
	Positive	Negative	P value
Age group			
Less than 5 years	56 (24.47 %)	61 (37.50 %)	0.000
6–15 years	88 (41.49 %)	39 (21.32 %)	
More than 15 years	74 (34.04 %)	66 (41.18 %)	
Sex			
Male	119 (57.98 %)	65 (40.44 %)	0.002
Female	79 (42.02 %)	91 (59.56 %)	
Occupation			
Farmers	158 (78.72 %)	111 (74.26 %)	0.054
Student/Teacher	44 (18.09 %)	32 (16.18 %)	
House wife	16 (03.19 %)	23 (09.56 %)	
Education			
None	121 (59.04 %)	89 (58.09 %)	0.579
Primary	30 (10.64 %)	22 (08.82 %)	
Secondary	26 (08.51 %)	18 (05.88 %)	
Arabic	51 (21.81 %)	47 (27.21%)	
Fever (Temp >37.5 °C)			
Yes	142 (70.21 %)	91 (59.56 %)	0.046
No	66 (29.79 %)	65 (40.44 %)	
Headache			
Yes	184 (92.55%)	115 (77.21 %)	0.000
No	24 (07.45%)	41 (22.79 %)	
Sweating			
Yes	57 (25.00 %)	31 (15.44 %)	0.037
No	151 (75.00 %)	125 (84.56 %)	
Chills and shivering			
Yes	89 (42.02 %)	53 (31.62 %)	0.056
No	119 (57.98 %)	103 (68.38 %)	
Nausea and vomiting			
Yes	134 (65.96 %)	35 (18.38 %)	0.002
No	74 (34.04 %)	121 (81.62 %)	
N	408 (58.02 %)	356 (41.98 %)	764

It was seen that malaria infection in males (57.98 %) was more common as compared to females (42.02 %) and many were within the 6–15 years age group (41.49 %). Most of the study participant were farmers among them 78.72 % were tested malaria positive. Almost half of the study participant tested malaria positive (59.04 %) were illiterate, and only 10.64 % of the study participant had completed primary school and 8.51 % had attended secondary school.

The analysis of symptoms showed that the occurrence of fever was notified among 70.21 % of the study participant diagnosed malaria positive. Most of the study participant 134 (65.96 %) reported nausea and vomiting. Other reported symptoms were headache 184 (92.55 %) cases, sweating in 57 (25.00 %), and chills and shivering in 89 (42.02 %). Fever, nausea/vomiting and headache were the predominant symptoms among the study participant with confirmed RDT’s in the Keur Soce health and demographic surveillance site during the 7 months of passive malaria surveillance.

### Global model using linear regression

The results showed that all the predictors returned VIF values are greater than 1.0 indicating that none of the variables are redundant. The explanatory variables-household size, housing materials, sleeping rooms, sheep and distance to breeding site returned significant t values of −0.25, 2.3, 4.39, 1.25 and 2.36 respectively. The OLS global model revealed that it explained about 70 % (adjusted R^2^ = 0.70) of the variation in malaria occurrence with AIC = 756.23 (Table 3). The ANOVA returned a significant F value = 13.83 and the Wald statistic has a significant Chi-squared value = 33.39. This means that generally, the model prove to be statistically significant. Jarque–Bera statistic returned a non-significant Chi-squared value = 2.12 (Table 4) indicating that the model’s prediction is free from bias (i.e. the residuals are normally distributed). The Chi-squared value (15.06) of the Koenker statistic is statistically significant. Importantly, it indicates relationship between some or perhaps all of the explanatory variables and the criterion variable are non-stationary or consistent across the study area. The explanation for this is that some independent variables may be important with respect to predicting the outcome of malaria in some villages, but in other villages may demonstrate weak predictive capability. It is evident that the model’s fitness will likely be improved with GWR (since the Koenker statistic detected non-stationarity in the relationship). This is because GWR assumes that relationships across space are non-static.

**Table 3:** Summary statistics for OLS

Variables	Coefficients value	Std. error	t statistic	P value	VIF
Intercept	304.8	2.453	0.58	0.456	
Household size	−0.02	0.044	−0.25	0.036*	1.804
Housing materials	0.56	0.221	2.3	0.005*	1.704
Village size	−0.06	0.068	−1.56	0.562	1.479
Sleeping rooms	2.12	0.024	4.39	0.003*	1.223
Bed net use	0.78	0.061	9.23	0.921	1.012
Distance to health Facilities	0.92	0.091	6.99	0.256	1.740
Sheep	0.15	0.051	1.25	0.001*	1.635
Distance to breeding site	0.43	0.014	2.36	0.003*	1.453

* Significant at 0.05

**Table 4:** OLS diagnostics statistics

Parameters	Value	P value
Joint F-statistic	13.83	0.00004*
Joint wald statistic	36.39	0.00013*
Koender statistic	15.06	0.01562*
Jarque–Bera statistic	2.12	0.04303*

R^2^ = 0.7696; Adj R^2^ = 0.70369; AIC = 756.23; AICc = 763.25

* Significant at 0.05

### Geographically-weighted regression

Geographically weighted regression resulted in a significantly better fit for all tested combinations of variables. Comparing both models with the AICc values, show that the value is reduced from 763.25 (for OLS model) to 679.5 (for GWR model) (Table 5). The GWR model was an improvement over the global. The difference between Adj R^2^ from the global and GWR is about 10 percent. This is a high percentage explained value not accounted for by the global model. Verifying with autocorrelation statistic (Moran’s I) returned a randomly distributed residuals with a z-score = − 1.14 and Moran index = − 0.14.

**Fig. 5:**
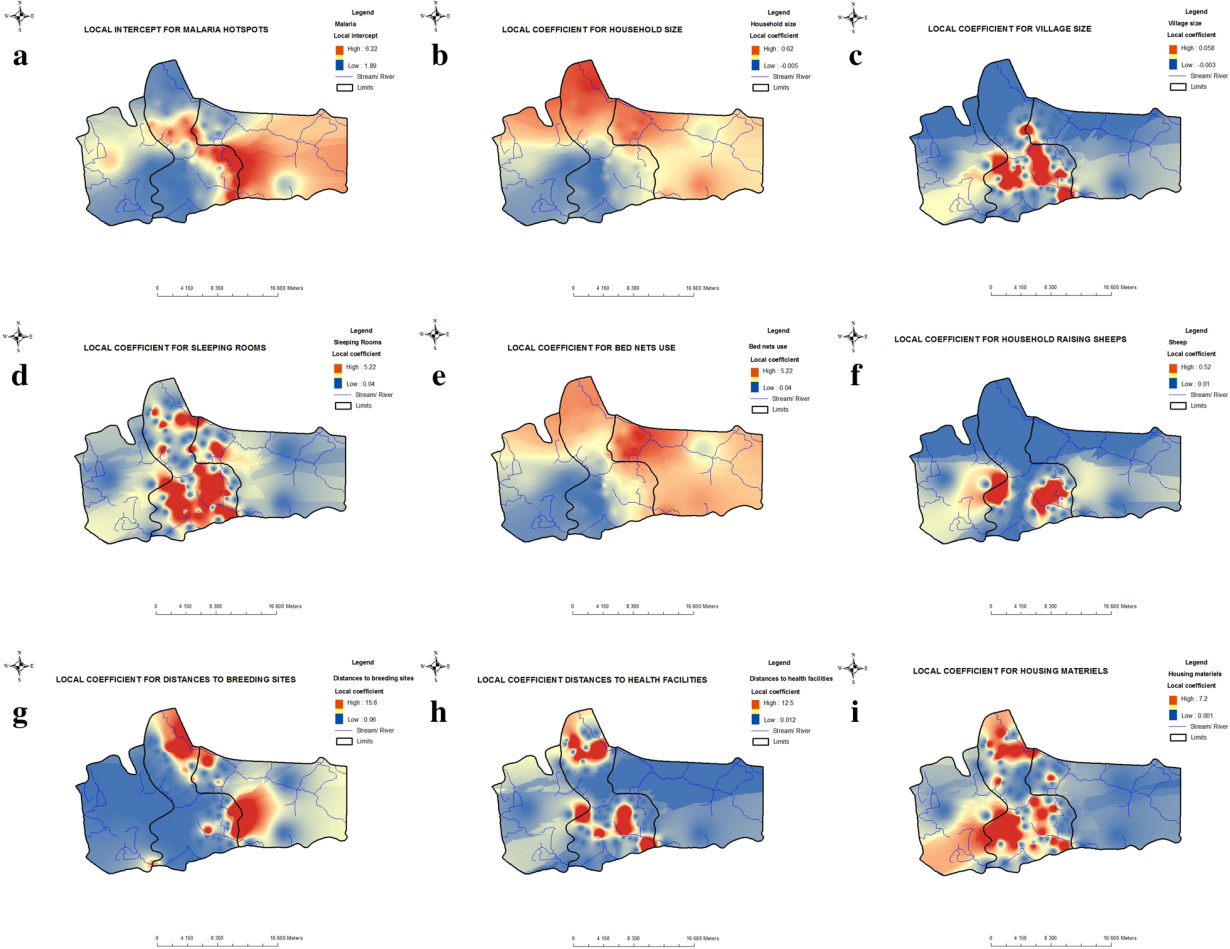
Local parameter estimates of GWR. **a** Local intercept for malaria hotspots (shows the spatial variation in the local intercept estimated by GWR). **b** Household size (indicates how malaria hotspots would change for each spatial unit change of the household size variable). **c** Village size (indicates how malaria hotspots would change for each spatial unit change of the village size variable). **d** Number of sleeping rooms (indicates how malaria hotspots would change for each spatial unit change of the number of sleeping rooms variable). **e** Bed net use (indicates how malaria hotspots would change for each spatial unit change of the bed net use variable). **f** Households raising sheep (indicates how malaria hotspots would change for each spatial unit change of the number of household raising sheep variable). **g** Distance to breeding sites (indicates how malaria hotspots would change for each spatial unit change of the distance to breeding sites variable). **h** Distance to health facilities (indicates how malaria hotspots would change for each spatial unit change of the distance to health facilities variable). **i** Housing materials (indicates how malaria hotspots would change for each spatial unit change of the housing materials variable)

The results from GWR allow to display and visualize the parameter estimates of each explanatory variable on a raster surface. This will make the complex relationship that varies over space easier to be understood. The resultant surface raster for the predictors show that there is spatial variation in relationship between sources of household water supply and cholera occurrence across the country (Fig. 5). Positive and negative relationships were manifested in the result of GWR. The positive relationship means that as specific explanatories variable increase, malaria cases equally reduce. On the other hand, negative relationship implies as specific explanatories variable increase, malaria cases equally increase. Local coefficient estimate for each explanatory variables are presented in Fig. 5. The colour ramp is graduated from light to dark gold. Areas with light shade represent areas where that particular variable exhibit strong influence on malaria occurrence while dark shade represent areas where that specific variable exhibit weak or low influence on malaria occurrence.

The geographically weighted regression on malaria hotspots resulted in local intercept ranging from 1.89 to 6.22 with a median of 3.5. Large positive values are distributed mainly in the south-east of the district where hotspots are more accurate while low values are mainly found in the centre and in the north Fig. 5a.

### Risk factors for malaria hotspots

Generally, OLS model was able to identify important variables that significantly explained the occurrence of malaria hostpots in Keur Soce HDSS. In the analysis of the risk factors, only these fundamental explanatory variables will be analysed in detail regarding the local coefficients derived from GWR model. Some predictors exhibited high spatial variability in the resultant parameter estimates of GWR model. In some cases, even contradicted the sign of global parameter estimates of OLS model. These predictors are village size and distance to health facilities, both reflected a combination of negative and positive coefficients across villages.

Among the explanatory variables, five are statistically significant; these are household size, housing materials, sleeping rooms, having sheep in the household and distance to breeding site. These variables are the most important with respect to explaining malaria hotspots. Housing materials and distance to nearest breeding site returned a highly significant *tvalue* (i.e. significant at 0.001). Drawing from this, there is 99 percent confidence that malaria occurrence in the study area positively influenced by these explanatories variables. This result is not unexpected because these factors can encourage the growth of *Anopheles* populations and facilitate the malaria transmission.

The results from the GWR shown that the risk of malaria hotpots occurrence increase from the north to the south. The household located at the north at more at risk compared to the household located at the south (Fig. 5b). The results had shown that for those household the size varied from 10 persons to 25 persons. The housing materials are also risk factors for malaria hotpots in Keur Soce. The highest coefficient are mainly found in the centre of the study area where most of the household are among the wealthy quintile (Fig. 5i).

## Discussion

Application of the spatial statistic methods successfully identified malaria clusters and clearly demonstrate malaria risk heterogeneity at local level. In the present study, the study described considerable spatial variation in malaria disease incidence and exposure to malaria-infected mosquitoes in an area of stable transmission intensity in Senegal. Clusters of high malaria incidence among study participant were interpreted as hotspots of malaria transmission. The distribution and level of malaria endemicity estimated in the analysis reveals significant spatial variation in malaria risk, which previous mapping studies failed to convey. The study also identified important clinical and modifiable socio-economic factors significantly associated with malaria risk, some of which with important operational relevance to the implementation of current malaria control strategies in the area. The survey results can be used to validate suggested malaria stratification schemes and improve the malaria control program’s targeting of interventions. The results from this study support the conclusion that malaria clusters may differ because of spatial variation [18] and that risks for malaria infection are associated with definable socio-demographic factors, which may be fundamental ecological units of malaria transmission [19]. A multitude of other factors may have an impact in these mostly rural settings, creating a context in which the impact of geographical factors and social behaviours on malaria prevalence and incidence may be particularly relevant. The results from the study support the fact that Distance to the nearest breeding site [7, 12, 14, 19–22], walling material [13], and household size [7] were independent predictors of living in a hotspot of malaria transmission. Proximity to breeding sites has been shown to increase the likelihood of exposure opportunities to mosquitoes and the results confirm that households closer to rivers are at increased risk of Plasmodium infection [23–27].

Household characteristics have also been shown to increase the likelihood of exposure opportunities to mosquitoes; for example, some studies have suggested an increased risk of malaria infection in houses made with vegetable material, which provides favourable conditions for mosquito survival [28–30].

**Table 5:** Model fitness comparison

Fitness parameters	OLS	GWR
AICc	763.25	679.5
R^2^	0.76	0.95
Adj R^2^	0.70	0.82

The association between vector density and environmental or climatic factors has been widely studied [12, 13, 31, 32] with rainfall and season consistently identified as significant factors while this study did not observe any association between climatic factors and malaria hotpots maybe due to the relative small variation in altitude.

A couple of studies have shown that increasing SES has a strong association with the malaria infection [29–32]. According to the WHO malaria report [33], malaria causes widespread premature death and suffering, imposing financial hardship on poor households, and holds back economic growth and improvements in living standards. Malaria flourishes in situations of social and environmental crisis, weak health systems and disadvantaged communities.

## Conclusion

In conclusion, malaria infection appears to be rare in Keur Soce health and demographic surveillance site while the transmission remains high during the rainy season. From the pure view of spatial extent, the hotspot analysis shows a strong spatial relationship of malaria occurrence in Keur Soce health and demographic surveillance site. A modelling based on GWR and OLS regression showed important risks factors of malaria hotspots. The outputs of such models can be a useful tool to understand occurrence of malaria hotspots in Senegal. An understanding of geographical variation and determination of the core areas of the disease may provide an explanation regarding possible proximal and distal contributors to malaria elimination in Senegal.
